# A Pervasive Healthcare System for COPD Patients

**DOI:** 10.3390/diagnostics9040135

**Published:** 2019-10-01

**Authors:** Hicham Ajami, Hamid Mcheick, Karam Mustapha

**Affiliations:** 1 Department of Computer Sciences and Mathematics, University of Québec at Chicoutimi, QC G7H 2B1, Canada; hamid_mcheick@uqac.ca; 2 Department of Mathematical and Industrial Engineering, Polytechnic of Montreal, Station centre-ville, Montreal, H3C 3A7, Canada; Karam.mustapha@gmail.com

**Keywords:** context-aware system, COPD, ontology, semantic web rule language (SWRL), healthcare systems

## Abstract

Chronic obstructive pulmonary disease (COPD) is one of the most severe public health problems worldwide. Pervasive computing technology creates a new opportunity to redesign the traditional pattern of medical system. While many pervasive healthcare systems are currently found in the literature, there is little published research on the effectiveness of these paradigms in the medical context. This paper designs and validates a rule-based ontology framework for COPD patients. Unlike conventional systems, this work presents a new vision of telemedicine and remote care solutions that will promote individual self-management and autonomy for COPD patients through an advanced decision-making technique. Rules accuracy estimates were 89% for monitoring vital signs, and environmental factors, and 87% for nutrition facts, and physical activities.

## 1. Introduction

COPD has a significant impact on individuals and society. Moreover, COPD represents an economic burden on the healthcare system. Statistics Canada [1], ranked COPD as the fifth leading cause of death in the country. Studies show that people with COPD are vulnerable to many natural events, environmental factors, and sudden worsening of any of the symptoms associated with this disease. Recent years have witnessed a widespread increase in the number of telemedicine projects. This kind of intervention can open a window onto the COPD patient’s life to assist with self-management and prevent declines. Telehealth refers to the remote monitoring and care of patients outside of the hospital setting. Typically, these systems are used with certain chronic diseases that are associated with frequent relapses. The early detection of worsening symptoms will help patients avoid severe problems and lengthy hospital stays [2]. The role of telemedicine in COPD is still being discussed. In 2018, Dr. Jean Bourbeau, a senior scientist at the research institute of McGill University Health Center, said that “telemedicine, both its application and results, is still controversial in COPD and the monitoring of physiological parameters does not solve the problem of predicting exacerbations that could lead to early therapy and prevention of hospital admissions” [3]. Recent studies cast serious doubts on the research findings in this domain [4,5,6]. 

However, pulmonologists still believe that telemonitoring can play an extremely important role if used properly. Brian Carlin, a pulmonary specialist and former chairman of the COPD Alliance, confirms that recognizing the main triggers in each patient is the best health protection plan to prevent flare-ups and thereby slow the progression of the disease; this is through maintaining an active lifestyle in a controlled environment without being exposed to such triggers. Specialists in this field suggested that “it may be more valuable to build the telemonitoring and telecommunication technology in the management of COPD on what we already know” [3]. This requires working on three different levels: first, find the safe range of environmental factors; second, adjust the normal limits of relevant biomarkers; and third, determine the external influences (e.g., food, excessive physical effort, climatic factors) on the patient’s body. The main contribution of this approach resides in the intelligent monitoring and control of persistent changes in the physiological parameters and the ambient environment. This work was part of a concentrated effort to create adaptive safe ranges for personalized biomarkers, where the normal values of these vital signs are often affected by the medical profile, the type of current exercise, the place, and the weather. Environmental factors are also one of the COPD irritants, where cumulative exposure to a multitude of climate hazards such as improper humidity levels or extreme weather temperatures, both indoor and outdoor air pollution, in addition to the abnormal concentrations of oxygen in the atmosphere, may threaten a patient’s lung health. Developing dynamic alarm thresholds is an important contribution because that would promote the services provided and increase the value of telemonitoring in self-management. Moreover, a customized threshold will help decrease the proportion of false alarms and differentiate between true exacerbation and normal variation. To achieve these goals, there is a need to develop a comprehensive representation of knowledge to capture the real context of the patient in order to avoid misdiagnosis and allow the dynamic reconfiguration of the health disorders threshold.

Rule-based ontology to support context-aware systems offers potential solutions to the multiscale nature of COPD. Many context-aware architectures have been proposed, but there is no published and validated research on the effectiveness of these computing paradigms within the context of COPD. In a previous work [7], we designed an ontological reasoning framework that provides a rules-driven, context-aware system for COPD patients. In this article, we will present the validation of that proposition, and demonstrating its efficiency through simulated examples of real-life scenarios and empirical data about the environment, activities, symptoms, and physiological parameters. For this purpose, we explain in detail the methods for extracting the medical rules of different contextual events. The paper examines the normal ranges of vital parameters during different activities of daily living, and sets a threshold limit for the environmental conditions, whether indoors or outdoors, which is adapted to suit each patient’s medical profile.

The rest of the paper is organized as follows. In Section 2, we review the existing telemonitoring platforms and ontology-based models, especially those designed for chronic pulmonary disease, and show the weaknesses that make them non-viable. In Section 3, we review the steps involved in this research and highlight our vision to deal with COPD. This hypothesis is based on comprehensive ontology and medical rules. In Section 4, we explain how we extract the SWRL rules using data analysis. In Section 5, we discuss the data collection process, then describe our implementation in detail and provide a performance analysis and results in Section 6, Section 7, and Section 8. Finally, the work is concluded in Section 9.

## 2. Related Works

For almost two decades now, the use of medical ontologies has no longer been limited to defining medical terminologies such as the systematized nomenclature of medicine—clinical terms (SNOMED CT) or the unified medical language system (UMLS), but has also become one of the most powerful solutions for tackling serious health problems and supporting the management of large amounts of complex data. Ontologies have also been used in hundreds of research projects concerned with medical issues such as diagnosis, self-management, and treatment [8,9,10,11,12].The ontological approach proved its effectiveness in the remote healthcare arena; for instance, Lasierra [13] and Rubio et al. [14] have presented robust examples of ontology usage in the telemonitoring domain for generic and specific chronic diseases. Lasierra proposed an autonomic computing ontology for integrated management at home using medical sensors. Rubio provides a formal representation of knowledge to describe the effect of technological context variations on clinical data quality and its impact on a patient’s treatment. Another example can be found in [15]: Benyahia et al. developed a generic ontology for monitoring patients diagnosed with chronic diseases. The proposed architecture aims to detect any anomalies or dangerous situations by collecting physiological and lifestyle data. Hristoskova et al. [16] presented an ontology-based ambient intelligence framework that supports real-time physiological monitoring of patients suffering from congestive heart failure. Ryu et al. [17] proposed a ubiquitous healthcare context model using an ontology; the model extracts the contextual information for implementing the healthcare service, taking into consideration the medical references and environments. Jong et al. [18] has designed an interactive healthcare system with wearable sensors that provides personalized services with formal ontology-driven specifications. In the same setting, an ontology-based context-aware framework for customized care has been presented by Ko et al. [19] as a form of wearable biomedical technology. An interesting projection of ontology in this domain can be found in [20], in which the authors built a context-aware mobile service aiming at supporting mobile caregivers and sharing information to improve the quality of life of people living with chronic diseases.

In addition to this obvious interest in ontology, most healthcare projects related to computer-assisted medical decision-making are often modeled using rule-based approaches. Semantic web rule language (SWRL) has emerged over existing W3C web ontology language (OWL) axioms to promote the expressiveness of the semantic web. The combination of OWL and SWRL specifications provides further inference capabilities beyond the inductive classification of description logics, with 78 built-in functions categorized across the comparisons, mathematics, Boolean values, strings, date, time and duration, URIs, and lists [21]. In the medical environment, there are several uses of rules; for example, if‒then rules can be used for chaining or mapping ontologies properties to achieve knowledge integration. By applying rules, the pattern of behaviors of all entities can be expressed, which would produce new facts and tailored services. Some examples of the incorporation of rules in healthcare ontologies as an essential component of decision support applications can be found in [22,23,24,25]. These rules are written in specific terms to infer useful information and then provide personalized care services to chronic patients according to their situations. For example, [22] established a set of predefined rules to trigger alarms when critical threshold levels are exceeded, while [23,24,25] used ontology-based rules for ubiquitous computing that allow for monitoring health anytime and anywhere. Furthermore, a few research projects have studied the use of SWRL to aid in diagnosis. These include [26] and [27], which provided rule-based ontologies to diagnose heart diseases and diabetes, respectively.

The use of ontology in COPD is only restricted to certain aspects of patients’ lives [8,11]. For example, the authors of [12] developed an ontology inspired by the autonomic computing paradigm that provides configurable services to support home-based care. The authors of [14] proposed a predictive model to extract relevant attributes and enable the early detection of deteriorations, but the proposed ontology aims at describing the basic structure of the application. Although a significant amount of research has been done to assess the importance of telehealth in COPD, the concept of integrated care services is still in its infancy. The use of semantic mapping between the physiological parameters, environmental factors, symptoms, physical activity, and patient-specific data to construct a telemonitoring system for COPD using ontologies was not found in the literature. This work will be the first building block for creating a comprehensive primary e-healthcare delivery system, capable of organizing various daily life scenarios for COPD patients in a healthy and safe environment.

## 3. Context-Aware System

Pervasive computing is considered one of the most impactful scientific achievements of the last decade. This conception created a revolution in end-user interactions through the concept of context awareness. Pervasive computing offers a new opportunity to redesign the pattern of conventional solutions as it can easily tailor its processes based on existing contextual situations. Many theoretical architectures have been proposed to enable context-aware computing in pervasive settings, especially in the healthcare domain. The overall architecture of context-aware systems and development process can be found in [7,28]. In this work, we distinguish four main components: context acquisition, representation, reasoning, and application. Context acquisition functions allow for querying physical devices to obtain contextual data. Given the various characteristics of contextual information such as heterogeneity, dynamicity, and imperfection, it is essential to define a model to describe these data. There are three main types of approaches to represent the context: the key-value approach, the object-oriented approach, and the ontology-based approach. On top of the context information, reasoning schemes are implemented to develop applications and services for particular needs.

In this work, an ontology-driven rules base for an expert medical system is described and applied. The proposed system provides an intelligent monitoring infrastructure to keep track of the physical status of patients, suggest recommendations, and deliver interventions in a timely manner.

**Figure 1:**
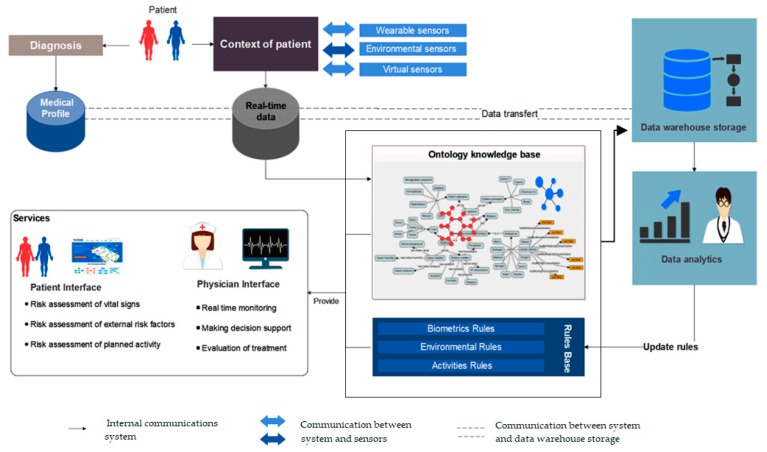
General architecture.

This process involves observing and controlling the behavior of physiological parameters and the surrounding environment. Consequently, the system adapts the safe ranges for the vital signs in proportion to demographic factors, medical profile, physical activity, and external ambiance. The presented project demonstrated its benefits not only in terms of real-time responses, but also in predicting the body’s changes in advance based on the effects of potential triggers. Due to the complexity of this domain of knowledge, we established a scenario-based approach to coordinate the evolution of such a decision-making system. The construction of scenarios is governed by executing a sequential modular strategy and evidence-based rules. This architecture is designed and implemented in four distinct layers: the acquisition layer is dedicated to collecting and properly transmitting different sorts of data, such as the medical profile of the COPD patient, biomarkers, and environmental information, whether gathered from wearable or fixed monitoring sensors. The semantic layer or the ontological schema has been used to interpret complex information and translate the real context of the patient into machine-understandable and accessible language. The generic representation of the semantic layer consists of a set of interdependent ontologies and many concepts related to the pulmonary disease, environment, devices, personal and medical information of the patient [3]. At the macro level, the telemonitoring system aims to detect all the possible hazardous events that could influence COPD patient. Since OWL has expressivity limitations on representing many types of contextual information, especially if‒then statements, our ontologies have been extended with forward-chaining rules. These rules were expressed in the semantic web rule language (SWRL) to describe all implications and consequences. The proposed rules are extracted from data analysis, existing medical guidelines, and the opinions of pneumologists. Practically, these rules are used by an inference engine to derive new facts, detect events, and predict potential risks. The novelty of these rules lies in the dynamic structure, which has the capacity to configure and reconfigure the secure boundaries according to the current circumstances and contexts. Figure 1 provides an explanation of the relationships between the constituent entities of the system in a simplified manner. The operational environment of this system is divided into four main parts. The first part is the collection of data. These data are distributed between the patient’s medical profile, obtained at the diagnostic stage, and the contextual information that we can obtain from virtual and real sensors. All this information must be kept in customized databases and kept consistent with the purpose, whether in the form of electronic medical records or conventional databases to manage the real-time context. The second part is the leading core of this system, where all the interpretation processes are carried out. This part is an ontology knowledge base, backed by complex medical rules that aim to draw a semi-complete representation of the patient’s life scenarios in a semantic programming language. In practice, the first and second parts are highly intertwined, so the collected data serve as input to the ontology, which in turn extracts the existing scenario and applies the rules of protection to ensure that patients are notified early on of any potential risk-related events.

The third part is related to system functions; the services offered by the system are divided into two main categories: (1) patient services, which include assessment of the vital signs, evaluation of the external risk factors, and estimation of the overall effects of activities. (2) caregivers’ services, which are supposed to provide multi-task interfaces for real-time monitoring, decision-making, and treatment evaluation. The last part controls all background processes. Dynamicity is one of the main features of this system. It develops its facts continuously since it sends raw and derived information periodically to the data warehouses for further analysis. This cycle allows the system to update its rules and promote personalized health services. In the next section, we will learn about the methods of extracting the medical rules of COPD.

## 4. Rules Extraction

In preparation for rule extraction, we performed a data analysis, reviewed the medical guidelines, interviewed experts, and examined published sources to map biomarkers of COPD patient to various real-life patterns. A rule is a description of how a patient is affected by internal bodily characteristics and external environmental factors. Obtaining medical rules from existing resources involves information extraction, analysis, filtering unwanted data, and refining ranges of values.

### 4.1. Vital Signs Rules

In clinical therapy, each patient must be recognized as an individual with a unique health state. However, grouping patients with a similar medical profile is an excellent solution to treat diseases. Unlike previous works and based on this scientific principle, we studied all relevant factors according to the personal medical profile, which includes the demographic information and the clinical chart of patients.

Hurst et al. [29] and Rajeh et al. [30] identified the main physiological parameters and symptoms to be monitored. Understanding the maximum possible extent of change for each variable in different scenarios is crucial for the early detection of pulmonary exacerbations. In the next section, we will try to find out how these parameters change with COPD patients in different medical profiles and during common daily life activities. Profile differences help to explain the discrepancies in medical care received by COPD patients. Having considered the guidelines, it is recommended that we divide the population of patients into groups or quartiles according to age, gender, stage, Body Mass Index (BMI), smoker, medication, and comorbidity. Identifying factors that may indicate if something is wrong with COPD patient is about to happen was a daunting task and took a long time. This study establishes 11 physical parameters that must be monitored namely, body temperature, blood pressure, heart rate, partial pressure oxygen (PaO_2_), oxygen saturation (SpO_2_), partial pressure carbon dioxide (PaCO_2_), oxygen consumption (VO_2_), respiration rate, blood pH, bicarbonate HCO_3_, and FEV1. Understanding the role of these biomarkers and their normal ranges in stable COPD patients in all potential scenarios grants us the ability to sense imminent danger. Patients were separated horizontally by gender, age, and stage; these groups have then been reclassified vertically according to the effects of BMI, smoking, medication, and comorbidities on the vital signs. To illustrate this point, we will provide an example to explain changes in heart rate with different profiles.

#### Heart Rate

**Figure 2:**
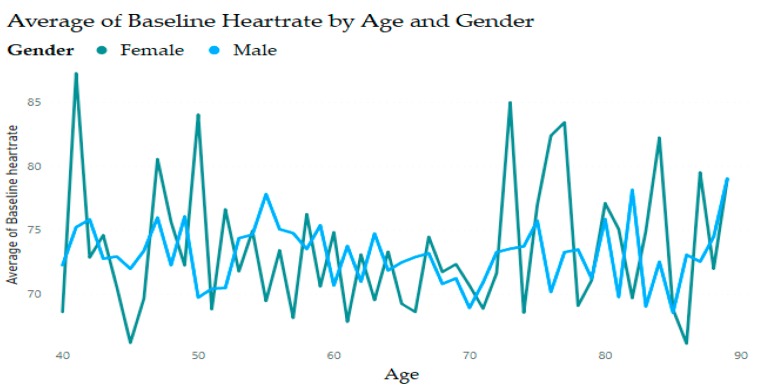
Heart rates by age and gender.

**Figure 3:**
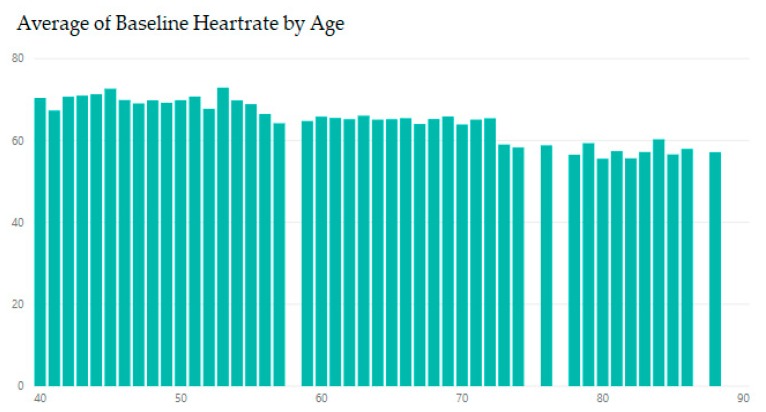
Heart rates by age.

Analysis of data obtained from medical records [31,32,33] finds that heart rate in women can be slightly different than in men. Figure 2 shows that females have a higher heart rate compared with males at all ages; this difference increases in early middle age and decreases in late adulthood. In contrast, the overall analysis shows a remarkable decline in the normal resting heart rate (Figure 3).

In the same context and in order to assess whether the expected effect of age truly has a powerful influence on changing heart rate variability, we designed the following controlling analysis paradigm. We sampled 1370 male patients aged 40 to 90 and assessed all the main characteristics such as COPD stage, gender, BMI, and comorbidities. For male patients in stage I, the results were as follows: 72.6 ± 12 beats/min for those aged 40‒50 years; 69.2 ± 11 beats/min for those aged between 50‒60 years; 68.70 ± 9 beats/min and 69.0 ± 10 beats/min for patients aged between 60‒70 and 70‒80, respectively; and 67.20 ± 9 beats/min for patients who were over the age of 80 (see Table 1).

In contrast, resting heart rate increased with the severity of COPD (*p* < 0.005), and we can easily notice that the resting heart rate of people aged 40 to 50 rose from 72.6 ± 12 in stage I to 84.9 ± 14 in stage IV. The analysis demonstrated that smoking increases the baseline heartbeat at a rate ranging from 6 to 10 beats/min. The values provided for smokers represent the mean heart rate at rest and the differences between sample proportions in the same conditions for male patients without specifications of age, BMI, etc. Resting heart rate was also associated with both obesity and the use of inhaler medication across all stages of COPD (*p* < 0.004 and *p* < 0.003). COPD patients tend to have more health problems, and COPD is frequently associated with a range of diseases such as congestive heart failure (CHF), coronary artery disease (CAD) or ischemic heart disease (IHD), pulmonary hypertension (PH), gastroesophageal reflux disease (GERD), high blood pressure (HBP), asthma, and anemia. The change in heart rate was only associated with four comorbidities, CHF, anemia, PH, and asthma, while HBP, IHD, and GERD were not statistically significant. One of the key factors that changes heart rate is activity. Heart rate differs from person to person during exercise or when doing any physical effort; this variation is determined by mathematical equations with an acceptable degree of accuracy. Activity would be classified into four categories: the first is sedentary, which requires the least amount of effort, or in other words, the minimal rate of oxygen consumption (e.g., sleeping, sitting, or lying down), the second category is called light-intensity activities (e.g., walking slowly, eating, preparing food, showering); the third category is defined as moderate physical activity (e.g., walking briskly, gardening, household cleaning); and the fourth category is the vigorous intensity level (e.g., jogging/running, swimming, walking upstairs, sports, carrying a heavy load).

In summary, this evidence-based analysis proves that heart rate varies depending on the medical profile of the patient, which in turn will affect the stable ranges of this vital sign during physical activities. The same analytical methodology has been used on the other physiological parameters mentioned above.

### 4.2. Indoor Rules

**Figure 4:**
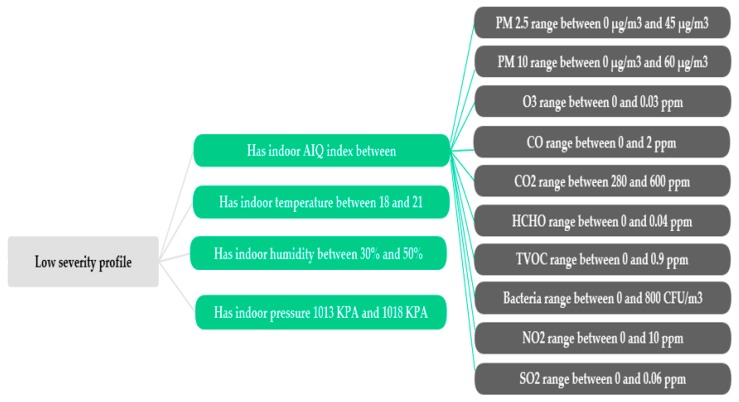
An example of indoor rules with low severity profile.

According to the Healthy Environments and Consumer Safety Branch (HECSB), Canadians spend approximately 90% of their life indoors [34], often due to the extreme nature of the climate. Therefore, it is very important to pay attention to the quality of that indoor air, temperature, humidity, and pressure, especially as COPD patients must live in a safe environment, away from any kind of irritants. Based on the outcomes of a wide range of studies [35,36,37,38,39,40,41,42,43,44,45,46,47] that addressed internal environmental conditions, we were able to draw up safe limits for all indoor climate factors. The foundations of internal protection have been translated into a hierarchical tree, as shown in Figure 4. In some cases, the indoor thresholds defined as comfortable have no significant correlation with the medical profile of the patient, while in other cases these thresholds were highly correlated with the severity of the patient profile. Reviewing all these factors in detail requires a lot of space, so we will simply list some of the existing findings.

Osman et al. [35] conducted an experiment on a sample of 254 participants; the mean age of patients was 69 years, with SD = 8.2; 45% of them were male. The sample included mild, moderate, and severe stages; smokers and non-smokers. Osman aimed at exploring whether the health status of COPD patients was associated with the recommended standards of indoor temperatures. This study demonstrated that the optimal respiratory recordings were obtained at and above 21 °C in the living room and at least 9 h at 18 °C in the bedroom during the night. Mu et al. [36] found that indoor temperature should be kept to an average of 18.2 °C. The WHO’s annual world health statistics report recommends that for certain groups such as people over the age of 65, the minimum indoor warmth threshold is 20 °C, but there is no evidence that these findings are applicable to chronic pulmonary diseases [37,38]. The COPD Foundation’s Slim Skinny Reference Guide (SSRG) [39] and Excellus BlueCross BlueShield [40] suggested keeping the indoor humidity below 40% and above 30%, respectively, to avoid harmful influences. Indoor pressure has a significant effect on breathing; it has been shown that negative pressure indoors may introduce harmful pollutants [41,42]. Generally, positive air pressure means that the indoor space is supplemented with 5 Pa/0.02 WC of filtered air with respect to the outside atmospheric pressure [43]. In multiple rooms, the most sensitive areas or places where the patient stays for a long time during the day should be the most highly pressurized. However, Ansley [44] indicated in an indirect manner that air pressure inside the chamber could be maintained at sea level. The standard sea-level pressure, by definition, equals 101.3 kPa = 760 mmHg [45].

Air pollution is considered to be one of the common triggers of exacerbations. Sometimes, pollutant levels in houses may be tens of times higher than the guidelines for outdoor air quality. Therefore, indoor air quality is a concern and must be monitored around the clock. Wang et al. [46] developed an indoor air quality index (IAQI) system based on the health risk assessment. The proposed IAQI is similar to the air quality index (AQI) developed by the U.S. Environmental Protection Agency (EPA). In this research, Wang suggested setting the health protection threshold of indoor air quality to 150 points for people with respiratory illnesses. In the same context, Saad et al. [47] presented another indoor environmental index. The IAQI value and status are divided into four categories: good (100‒76), normal (75‒51), unhealthy (50‒26), and hazardous (25‒0). Saad mentioned in his project that only good and moderate levels are appropriate for sensitive groups of people. The regulations for safe indoor air quality in these two indicators are almost identical, and we will adopt the most stringent index in case of different values. Indoor air quality is generally assessed by separately measuring major air pollutants such as PM10, PM2.5, O_3_, CO, CO_2_, HCHO, TVOC, bacteria, fungi, NO_2_, and SO_2_.

### 4.3. Outdoor Rules

There are several reasons why healthy outdoor environments are important for COPD patients. Unlike the internal environment, we cannot control the external environmental factors. There are six main factors that affect the patient: outdoor temperature, humidity, wind speed, precipitation, atmospheric pressure, and air quality. As with physiological parameters and internal environmental conditions, and considering space constraints, we will try to give a clear picture of how to select safe external environmental ranges.

**Figure 5:**
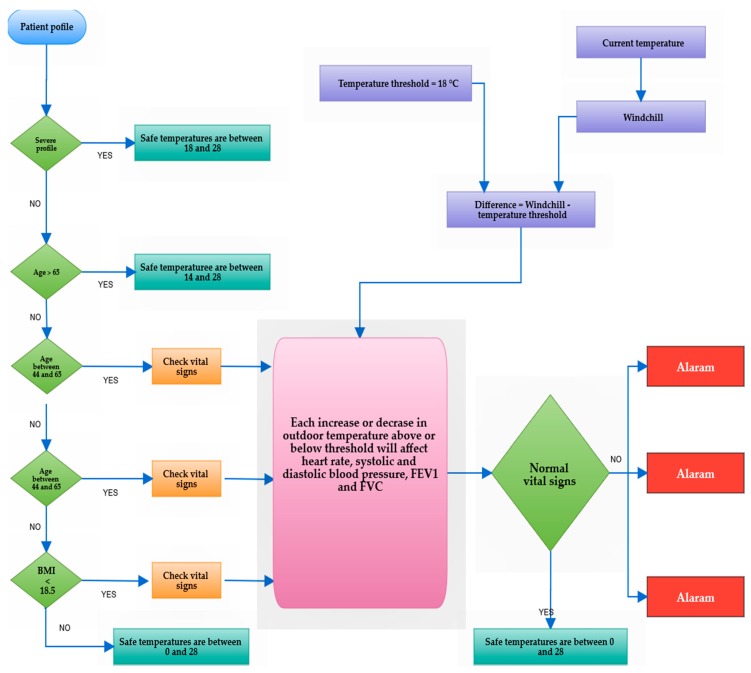
Outdoor temperature rules.

In research and clinical trials provided by [35,48,49,50,51,52,53,54], respiratory problems and exacerbations was highly correlated to the outdoor temperature. For example, Madaniyazi et al. [52] investigated the relationship between variations in heart rate (HR) and blood pressure (BP), including systolic blood pressure and diastolic blood pressure, and seasonal changes in ambient temperature. This work classified participants based on a set of physical and social characteristics such as age, sex, BMI, habits, physical activity, dietary habits, income, education level, work type, and medical history. The analysis concluded that cold and hot weather had an evident impact on HR and BP, but in different proportions according to the individual information of each patient. In general, for prolonged exposure to cold weather in the total population, a 1 °C decrease of mean temperature below the threshold temperature (which was estimated to be 22 and 27 °C for HR and BP, respectively) was linked to a 0.063 beats/min increase in HR, a 0.129 mmHg increase in SBP, and a 0.065 mmHg increase in DBP. On the other hand, hot weather was positively correlated with heart rate and blood pressure, a 1 °C increase in mean temperature above the threshold (previously mentioned) was associated with a 0.133 beats/min increase in HR, a 0.605 mmHg increase in SBP, and a 0.128 mmHg increase in DBP [52]. In the same context, Donaldson et al. [53] studied the effect of temperature on lung function and symptoms in COPD. The authors found that forced expiratory volume in one second (FEV1) and forced vital capacity (FVC) fell remarkably by a median of 44.9 mL (at 2.20 mL·°C^−1^ outdoors and range: -113,-229 mL) and 74.2 mL (at 3.64 mL·°C^−1^ outdoors and range -454,-991 mL), respectively, between the warmest weeks, with a mean temperature of 21.1 °C and coldest weeks with a mean temperature of 0.78 °C. The size of the effect was somewhat similar in the research done by [54], with a 0.71% and 0.59% decrease of FEV1 for every 10 °F increase in mean annual ambient temperature, which is estimated to be around 57 °F in two cohort studies. The flowchart in Figure 5 gives a summary of these extended reflections related to the outdoor temperature.

The environmental air consists of approximately 78% nitrogen (N_2_), 21% oxygen (O_2_), and 1% other gases, 0.038% of them for carbon dioxide (CO_2_) [55,56,57]. At sea level, where the standard barometric pressure is 760 mmHg, the estimated partial pressures of these three gases can be 593 mmHg for N_2_, 160 mmHg for O_2_, and 0.02 mmHg for CO_2_ [55]. At high altitude, the atmospheric pressure that is acting on the air gases is significantly less than the ordinary pressure at sea level. Therefore, the oxygen molecules in the air are spread further apart, reducing the oxygen concentration in the air and thus reducing the oxygen saturation in the blood [58], which can pose risks to people with COPD. There are several references describing the ratios of both oxygen and carbon dioxide with the change in altitude, temperature, and pressure [59]. These reference charts help us to develop the first layer of protection rules.

**Figure 6:**
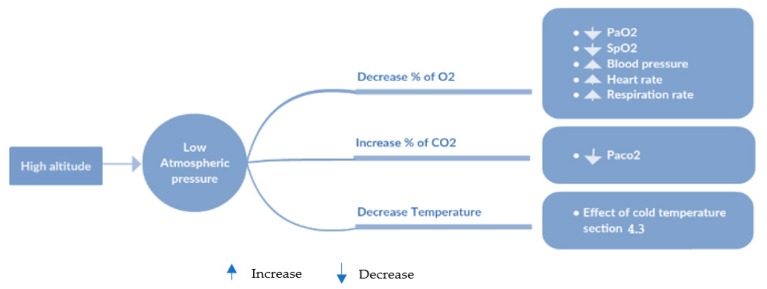
The effect of atmospheric pressure.

**Figure 7:**
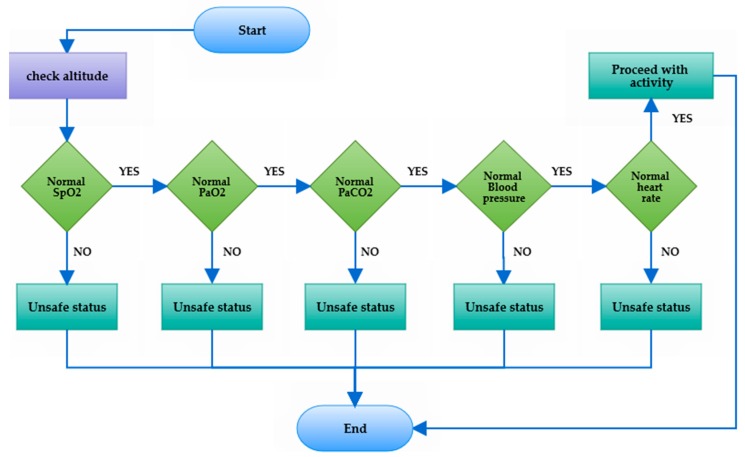
A simple flowchart of altitude and biomarker rules.

The barometric and hypsometric formula for altitudes between 0 km and 11 km is depicted in the next equation: P = 760 × (1 − H_b_/4430.76923)^5.255876^ provided by [60], where the pressure P and the altitude H_b_ are expressed in mmHg and m, respectively. The analysis of physiological responses to extreme atmospheric pressure revealed the presence of a significant direct relationship. In a meta-regression study, Mingi et al. [61] have shown an incremental increase in the prevalence of hypertension by 2% with every 100 m rise in altitude. This higher pressure means that the heart must work harder to pump blood through the arteries [62]. At the same time, exposure to low barometric pressure must result in a decrease in the PaO_2_ in alveoli, leading to reduced oxygen blood saturation (SpO_2_) [55,63]. Given the varying severity of COPD, Dillard et al. [64], developed equations to estimate PaO_2_ at high altitude, incorporating PaO_2_ on the ground and FEV1% predicted. The partial pressure of oxygen in the COPD patient’s arterial blood (PaO_2_) with baseline FEV1 below 1.5 L should be evaluated prior to high-altitude travel to determine whether the lung conditions require supplemental oxygen based on the regression equation provided by Dillard [64]: *P_a_O_2 altitude_* = (0.5196 × *P_a_O_2 sea level_*) + (11.856 × *FEV1*) – 1.76. Netzer et al. [65] verified the findings related to fluctuations of SpO_2_ and heart rate during a mountain hike; the authors found that SpO_2_ values are significantly lower and heart rate values are significantly higher in a high-altitude environment. In the same context, Maldonado et al. [66] assessed the risks in the highlands through comparing the effects of the lower barometric pressure on COPD patients at rest and during exercise. The study provided a detailed description of endurance time, inspiratory capacity, arterial blood gases, and lactate. Rules of barometric pressure at high altitude help patients to tell if it is safe for them to travel to intermediate altitude. Briefly, altitude exposure is not recommended for patients with severe COPD; however, these rules could minimize the risk of adverse effects, and determine the level of physical activity to ensure optimal health and prevent exacerbations. Based on this information, we developed an algorithm that evaluate the effect of low barometric pressure on air compounds (O_2_ and CO_2_) and air temperature, which are reflected in the vital signs of the body such as SpO_2_, heart rate, respiration rate, and PaCO_2_ (see Figure 6). The proposed rules check the altitude periodically or when needed to calculate the change in the biological parameters, as shown in Figure 7.

Research in the medical domain in recent years has confirmed that short- and long-term exposure to air pollutants increases the risk of COPD exacerbations [67,68]. Compliance with environmental regulations and standards decreases the levels of air pollution and reduces the exacerbation burden of COPD. Many countries and organizations are using the air quality index in assessing the concentrations of air pollutants. However, there is a lack of studies about the clinical adverse effects of outdoor air pollution on COPD patients. The pollutants in the surrounding air can be classified into primary and secondary types depending on the compounds and formation process. Several evidence-based policies and recommendations for air quality have been developed to reduce the impacts of pollution on human health and safety. In this work we adopted the various Canadian air quality objectives and standards: the Canadian National Ambient Air Quality Objectives [69], the standards for the atmospheric quality throughout the territory of Québec [70], the Ambient Air Quality Criteria (AAQCs) [71] developed by the Ontario Ministry of the Environment and Climate Change, and the British Columbia Ambient Air Quality Objective [72]. These standards helped us to create four levels of protection based on the profile of the patient.

Traditional information indicates that optimum humidity levels range from 30% to 60%, but Tseng [49] found that a lower humidity level, starting at around 34%, was positively associated with parameters related to an increase in COPD exacerbation and hospital admissions with more sunshine hours. A study in Hong Kong [73] supported this contention that dry air may aggravate symptoms, noting that the respiratory system of COPD patients seems to be protected by humidity contained in the air. Conversely, Freitas et al. [74] and Hayes et al. [75] demonstrated the indirect effects of high humidity and warm climate on respiratory and circulatory diseases. In this paper we developed a humidity‒temperature reference to control outdoor activities and set the maximum exposure time per 24 h.

### 4.4. Adaptation to Dynamic Context

**Figure 8:**
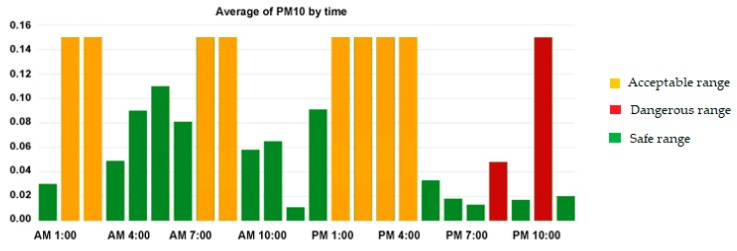
The safe levels of PM 10 over time.

With the growing need to adapt services to the patient’s context, we have added some vitality to the structure of the proposed rules. In this project, we used rules with dynamic threshold instead of predefined and static values. Many safe constants will not stay safe all the time. Prolonged and persistently high levels of biometrics are undesirable even if the captured values are normal to some extent. In contrast, some environmental variables may also have a negative effect if the exposure time exceeds the allowable limits or coincides with another influential factors (e.g., temperature, wind speed, and humidity). The boundaries of the safe zone are often spaced out, i.e., there is a wide margin between the high and low limits. The rules expand and minimize the safe area of humidity, heat, and air quality index automatically according to the exposure time and other related factors. For example, the safety threshold for PM10 is 0.150 mg/m^3^, but if a patient from the low-severity group stays in this room for more than 8 h, the safe zone limits will change to 0.020 mg/m^3^ (see Figure 8). This process has been accomplished with the help of pulmonary specialists.

This information aims at building a safe environment for COPD patients by converting this contextual schema into the SWRL rules base. These rules focus on evaluating body functions, environment information, calculating the severity profile, and outputting the right decisions.

## 5. Dataset

**Figure 9:**
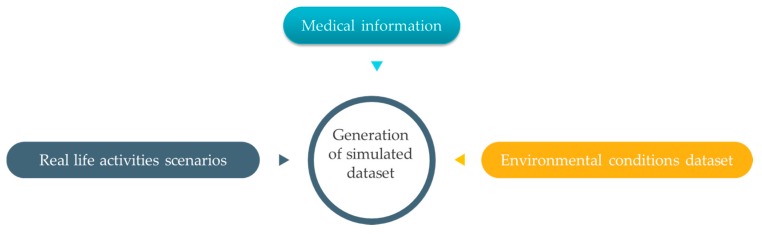
Dataset.

Gathering such different kinds of data from real sensors is subject to some practical limitations such as ethical approval, financial costs, and deployment time. Hence, researchers suggest an alternative experimental method using intelligent simulation. Based on this solution, each project can be proved operationally efficient before proceeding to the later stages of implementation. In this context, many simulation scenarios need to be performed to prove the feasibility of the proposed approach. These trials include placing COPD patients in different environmental conditions with multiple shapes of everyday life activities. Human health is a very sensitive topic and must be treated with high accuracy; this means that all created scenarios should provide a representation close to everyday-life experiences in a realistic environment in terms of the weather, pollution, and other surrounding factors. The second point we need to take into consideration when we generate data is the time scale of monitoring, where long-term scenarios allow the evaluation of the dynamicity and computing paradigms of the system. The third requirement in this operational context is to consider abnormal situations or irregular events that may occur during the monitoring of COPD patients. To create realistic and effective scenarios, it is necessary to carry out a simulation over a long period of time that involves the expected activities of patients and the environmental conditions that influence the health of people suffering from COPD. There are three basic data sources to build such scenarios: (1) medical information, (2) daily life activities, and (3) the environmental conditions, as shown in Figure 9.

### 5.1. Real-Life Activities

**Figure 10:**
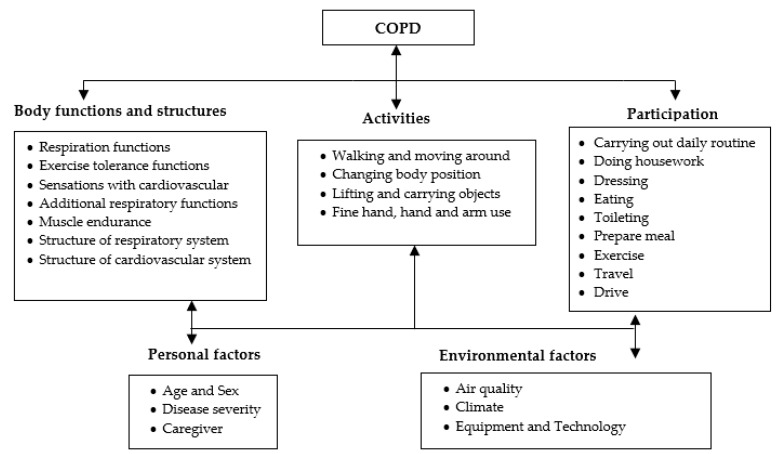
ICF model.

There are relatively many published studies about the simulation of activities; for instance, Elbayoudi et al. [76] have simulated human behavior in intelligent domestic space. Limousine et al. [77] proposed a grammatical approach to facilitate the representation of complex indoor human activities scenarios and consider the abnormal activities using a hierarchical hidden Markov model. In a similar work, Aritoni et al. [78] presented a generative model able to define the vast majority of daily routine events that would be of interest in a real-time monitoring system. Mshali et al. [79] used a novel strategy for generating long-term realistic scenarios. The suggested approach considers the person’s profile, the activities, and the logical relationships between these activities. Unfortunately, the publicly available datasets that simulate patients’ activities did not consider outdoor scenarios such as driving, walking outside, running, etc. They involve only a subset of the most common indoor actions. Based on these findings, we attempted to extend the existing indoor scenarios proposed by Mshali [79] using the same approach built upon Markovian models. Many other scenarios have been built upon Markovian models; we created new sequences of expected activities, including outdoor actions that can be performed by COPD patients. These scenarios took into consideration the levels of severity and disabilities, as provided for in the international classification of functioning (ICF). This is a classification of health domain and its main related aspects; it is intended to describe how patients live with their physical or mental illness [80,81]. According to the ICF model, COPD can influence the participation of patients in practical life, or daily living activities, depending on their health conditions. Figure 10 shows the ICF model, which could be adapted for each COPD patient. As depicted in Figure 10, the ICF framework presents the functioning and disability of a patient with COPD as mutualistic interactions among five different entities: body functions and structures, activities, participation, environmental, and personal factors.

Based on this framework, Bui [82] discussed the physical capacity for various body structures and functions of COPD patients, while Alda [83] studied the association between activities and participation components and the grades of patients’ airflow limitation. In the same context, Marilyn [84] showed the types of physical activities performed by COPD patients. All these facts were combined in one algorithm to extract a series of daily activities in accordance with the medical and personal profile of the patient in terms of demographic characteristics and physical ability.

These daily life activities of COPD patients have been divided into six successive sequences associated with six time periods from sunrise to sunset. Each of these sequences consists of a set of coordinated activities, with a random duration created through transition probabilities matrices. The matrix was filled in a systematic and deliberate manner, where high probability values were given to all the possible events that might occur during a specified period of the daytime, such as driving to work between 8:00 a.m. and 9:00 a.m. or having lunch between 2:00 p.m. and 4:00 p.m. The generated dataset comprises most of the main activities and their related actions that are deduced according to the patient’s ability to perform activities of daily living either with or without the help of others.

**Figure 11:**
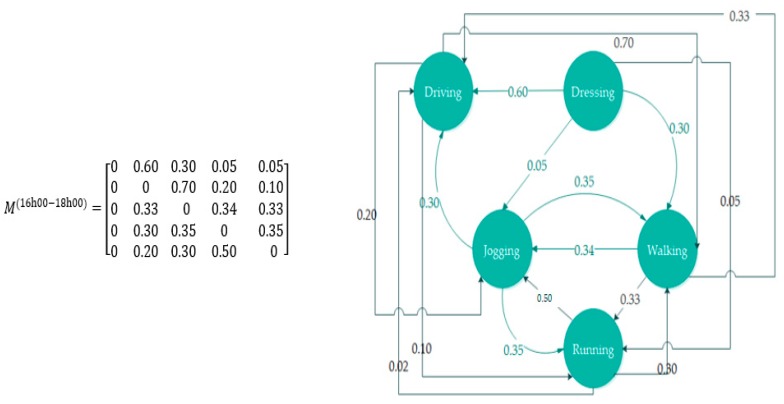
The matrix of transition probabilities.

In this project, 18 typical activities, describing the behaviors of COPD patients in public life, have been considered. Some of the indoor activities were taken from the proposed list in [85], while a range of activities was added based on the patient’s needs outside of the home setting. The activities selected are: eating, dressing, driving, walking, jogging, running, traveling, washing, toileting, housekeeping, laundry, cooking, telephone use, taking medication, watching TV, reading, and sleeping. Figure 11 provides a graphical interpretation of the probabilities of transitions followed to generate a random activities sequence within the given interval of time between 4:00 and 6:00 p.m. Moreover, for greater accuracy in the generation of the realistic scenarios, Mshali [79] added additional constraints that bound the frequency [*f_min_*, *f_max_*] of each activity and the total duration [*D_min_*, *D_max_*] of the sequence; for more details, see [85]. In addition to the previous restrictions, the process of building the data must consider the types of activities that the COPD patient can do, as stated in the ICT framework. In other words, adhering to a realistic representation requires respect for the constraints of the physical capacity profile of patients of whatever age or stage.

A small sample of the data is presented in Table 2.

### 5.2. Environmental Conditions

Assembling environmental data was easier, in that we found many open sources that describe the environmental conditions of indoor and outdoor spaces over long intervals. One of the most interesting outdoor datasets for environmental information was published by the Ministry of Environment and Climate Change Strategy in British Columbia, Canada [86]. These datasets contain continuous readings of meteorological and pollutant indexes from air quality monitoring stations across the province from 1980 until the end of 2017.

The Canadian environment has extreme climatic conditions that include unusual and unpredictable weather. The published dataset contains meteorological time series encompassing temperature in degrees Celsius, relative humidity in percent, wind speed in meters per second, wind direction in degrees from true north/azimuth, precipitation in millimeters, and barometric pressure in kilopascals. In addition to the meteorological data, the monitoring stations in British Columbia recorded the air quality health index, the level of carbon monoxide in parts per million, hydrogen sulfide and total reduced sulfur in ppb, nitric oxide in parts per billion, nitrogen dioxide in ppb, sulfur dioxide (SO_2_) in ppb, ground-level ozone in ppb, particulate matter with a diameter of 2.5 μm or less in micrograms per cubic metre (μg/m^3^), and particulate matter with a diameter of 10 μm or less in μg/m^3^. The dataset pertaining to environmental elements follows the form date, time, longitude, latitude, altitude, temperature, humidity, wind speed, wind direction, precipitation, barometric pressure, carbon monoxide, hydrogen sulfide, sulfur, nitric oxide, nitrogen dioxide, sulfur dioxide, ozone, PM 2.5, and PM 10. Some of the data is provided in Table 3 and Table 4.

Simulation of the internal environment is also important when the patient spends most of his/her time at home. The indoor data are tracked via GAMS environmental monitoring company, Shanghai [87]. The indoor air quality dataset contains temperature, humidity, carbon dioxide (CO_2_), volatile organic compounds (VOC), particulate matter with a diameter of 2.5 μm (PM2.5), and particulate matter with a diameter of 10 μm (PM10). The structure of the indoor environment dataset consists of date, time, indoor temperature, indoor humidity, CO_2_, VOC, PM 2.5, and PM10 (see Table 5).

### 5.3. Medical Profile

Medical profiles are vital in ensuring the validation process. In this context, we have collected thousands of electronic medical records, hospital admission data, and measures of outcome in clinical studies from different medical sources (MIR Clinic [32] and Al-Sahel Hospital [31]). As we can see in the Table 6, these datasets contain specific information about age, gender, weight, height, BMI, smoking, comorbidities, medications, the mMRC dyspnea scale, the stage of COPD, normal baseline vital signs such as body temperature, diastolic blood pressure, systolic blood pressure, heart rate, partial pressure oxygen (PaO_2_), oxygen saturation (SpO_2_), partial pressure carbon dioxide (PaCO_2_), oxygen consumption (VO_2_), respiration rate, pH, HCO_3_, and a spirometry test that includes FEV1, VC, FVC, FEV1/VC, FEV1/FVC, PEF, PEF2575, ELA, FET, FEF25%, FEF50%, FEF75%, EVol, MVVcalc, FIVC, FIV1 FIV1/FIVC, and PEF. These datasets contain an incremental cycle test (ICT) and a six-minute walking test (6MWT), which would give us a clear understanding of the upper and lower limits of the physiological parameters of patients during sedentary, light, moderate, and vigorous activity.

## 6. Implementation

As seen in the previous section, the experimental available datasets consist of values collected at different observational units. So, we synchronized the time scale between these data without affecting the accuracy, and mapped the simulated representation of the real-life activity with corresponding vital signs, according to the medical file. Therefore, the new dataset consists of three fully connected observations, the activities that have been generated using the MATLAB, the environmental conditions that are captured by real sensors, and the biomarkers that are obtained from medical sources. The simulated scenarios revolve around creating sequential records over a 12-month period for COPD patients with different levels of disease severity and autonomy. Each record is a description of the activities performed, the environmental factors, and the vital signs at a certain time. The objective of this process was to create a dataset of the COPD parameters associated with variation in the bodily activity and surrounding factors. The obtained databases consist of a total of 104 parameters, giving distinct advantages over previous studies even though the accuracy is unknown. For example, this dataset utilizes the best available environmental characteristics dataset for weather conditions and outdoor pollutants in Canada; it also uses real-indoor observations taken from a credible research project. The sequences of daily living activities were generated through Markov models and algorithms for probabilistic logic, while the medical profile has been issued by reliable medical sources. To prove the advantages of this work, we conducted many experimental simulations for the whole scenario of biological reactions to physical activities and external influences over 12 months in 100 virtual patients. First, we considered the scenarios of COPD patients having the same level of dependency but in different stages of the disease. Secondly, we simulated COPD in elderly patients who have lost some of their physical ability by excluding some kinds of autonomous activities of daily living tasks. Thirdly, for accuracy reasons, we created our simulations to compare the efficiency of this system during the winter, spring, summer, and autumn. We evaluated the performance of the proposed system for the identification of abnormal situations or patterns that may pose serious health risks for COPD patients. This evaluation consists of quantifying the level of computing or compliance with the expected time and the capacities of rules repository. COPD patients’ needs change according to their medical profiles and the type of hazards. The adaptation services with these changes are an essential point to test in such a remote healthcare system; therefore, it is of paramount importance to deliver a quick and accurate response to a sudden decline in vital parameters and general health status. Using a specific amount of data, the system categorizes the monitored conditions as either normal or abnormal. In the next section, we briefly describe the implementation steps of this project using Protégé that was developed by the Stanford Center for biomedical informatics research at the Stanford University School of Medicine. 

**Figure 12:**
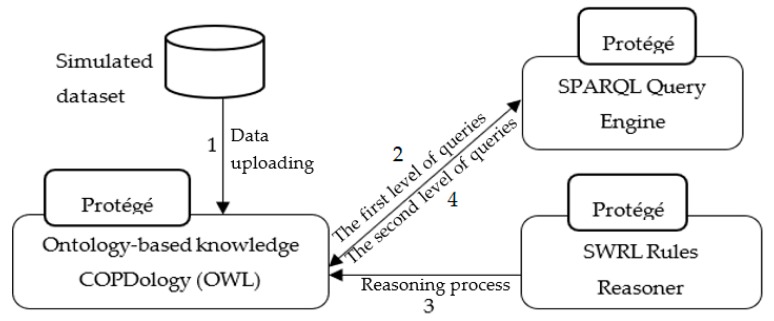
Flow in the implemented system.

The initial implementation presented in the Figure 12 is carried out in the following manner: (1) the simulated data, stored in Excel spreadsheet files, will be uploaded directly to Ontology-based knowledge using Cellfie plugin. (2) The SPARQL query engine accesses the Knowledge Base to retrieve information from a patient’s profile regarding current location and activity, etc. (3) The SWRL rule reasoner adds additional information such as normal ranges, and appropriate environment, to the Knowledge Base. Moreover, the SWRL rule reasoner performs reasoning on the updated Knowledge Base of COPD domain and newly inferred facts are added to the Knowledge Base. (4) The SPARQL query engine accesses the Knowledge Base to retrieve notifications and recommendations according to the patient’s context.

**Figure 13:**
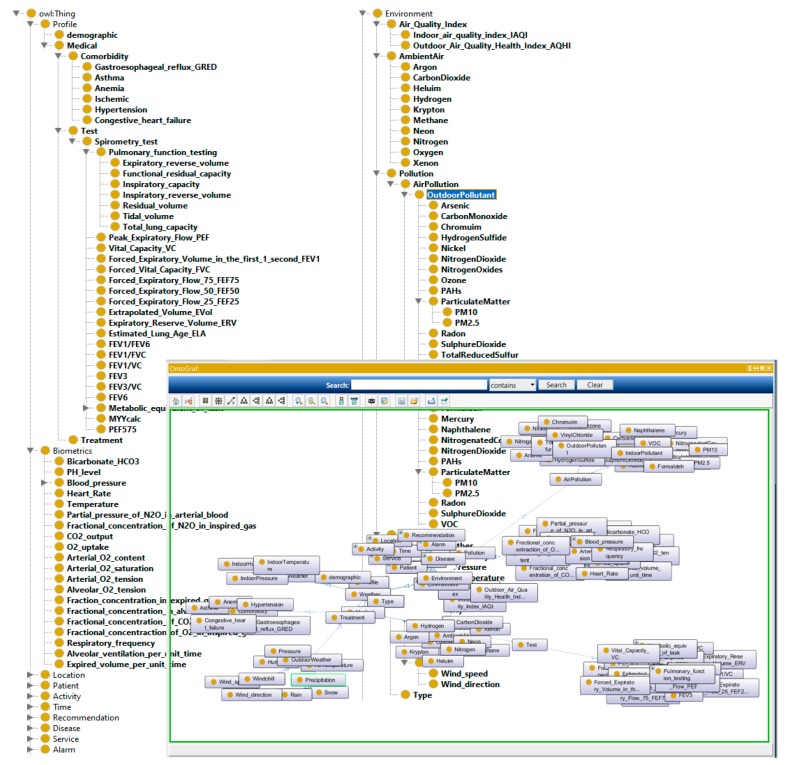
Portion of the proposed ontology.

We constructed eight ontologies for monitoring COPD patient and representing machine-understandable Knowledge Base. As shown in Figure 13, the developed ontologies consist of concepts related to the personal and medical profile, physical examinations, laboratory test, location, activity, environment, time, recommendations, and diseases.

In OWL, properties are used to describe relationships between individuals (instances) or to attribute an XML data type (such as string, integer, etc.) to individuals. There are two main types of properties, object properties that relate individuals from two classes, and datatype properties that are used to save real data values for the individuals of classes in a specific data format. Data type and object properties play a fundamental role in the ontology. Our COPDology consists of hundreds of object and data properties.

**Figure 14:**
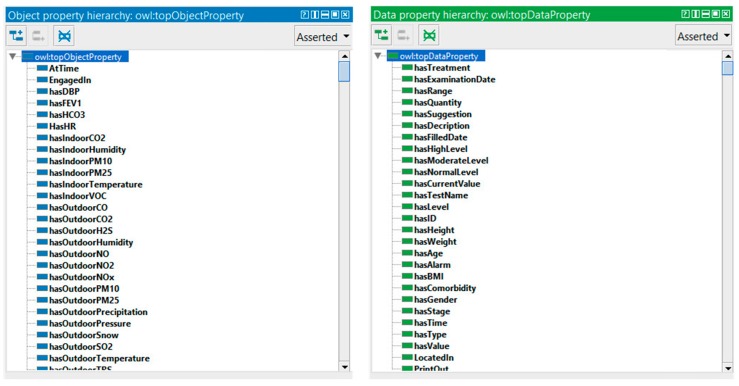
Part of datatype and object property.

These properties are used to define the profile of patient (e.g., hasAge, hasBMI, hasGender), to recognize location and activity (e.g., LocatedAt, EngagedIn), to characterize environment (e.g., hasIndoorTemperature, hasOutdoorPressure), or to trigger alarms and provide suggestions (e.g., hasAlarm, hasSuggestion). Figure 14 shows an example of the properties defined in our ontology.

**Figure 15:**
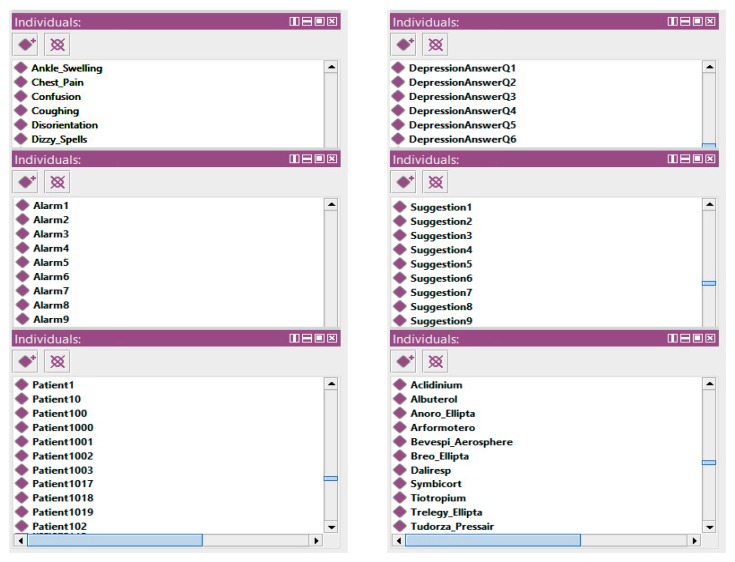
Examples of instances in the ontology.

The instances were instantiated automatically in Protégé from the gathered dataset. As mentioned before, the data simulates patient conditions and environment characteristics. These individuals can provide a list of medical records, profiles, physiological signs, safe thresholds, potential risks, recommendations, and explanations to the user when running the system. Figure 15 shows an example of instances created in the ontology. This example, from left to right, illustrates symptoms, answered questions, alarms, suggestions, patient identifier, and treatment.

**Figure 16:**
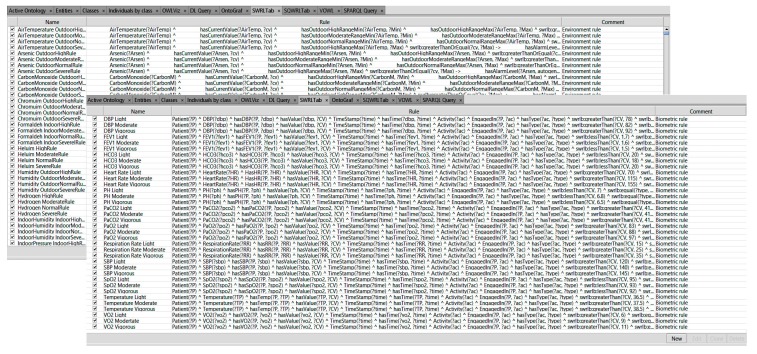
SWRL rules bases.

Thousands of SWRL rules were used to manage the status of almost 600 COPD medical profiles in various circumstances. Both chronic and multichronic patient profiles were configured by using the proposed rules. These configured profiles could be used to monitor the safe conditions of patients with chronic or multi chronic illnesses. Based on the medical information found in the guidelines and other information provided by physicians, we created 20,328 rules using forward chaining of inference. These rules use concepts/axioms defined in our COPDology. The rules are set to achieve different goals such as (i) verifying the profile of patients; (ii) detecting the location; (iii) evaluating the patient’s status and surrounding conditions; and (iv) providing the corresponding service for patients. For more details, please refer to the reasoning section of our previous work [7]. Figure 16 shows the list of SWRL rules that we created in the SWRL tab to monitor the patient.

The use of SWRL rules in combination with COPDology instances has been studied in this article to provide personalized care services. Having reasoning techniques of ontologies and rules that contain the asserted and inferred statements, we used SPARQL to retrieve and derive contextual information from the knowledge base. Although SPARQL is classified as a query language, it can be considered more than this since it provides different query forms that allow its functionalities to be extended. For example, SPARQL can provide functions to verify whether certain constraints currently hold in an RDF triple store (ASK), or to specify inference rules using (CONSTRUCT), not only, but SPARQL can also be used to perform DELETE or INSERT operations. In contrast, the simplest form of a basic graph pattern (WHERE) can establish complex patterns by using UNION, OPTIONAL, and FILTER clauses. Therefore, the same SPARQL rule engine can be a used to execute functions and clinical monitoring rules, providing great flexibility in the definition of both types of data. Furthermore, SPARQL has also been studied to complement OWL expressiveness for arithmetic functions that will be included in the COPDology. This functionality indicates how information can be mathematically processed. A total of 11 arithmetic functions (ABS, AVG, division, multiplication, cell, floor, count, min, max, round, and sum) have been used to compensate for the OWL limitations. The aim of these queries is to retrieve any information relating to an identified instance, such as a sign, symptom, treatment, alarm, recommendation, decision, etc. In this section, we will present some queries that could be used to display important information and illustrate the implementation of the main functions.

*Query 1: How to Show Patient, Age, Gender, Stage, Location, Activity, Vital Signs, Alarm, and Time*

**Figure 17:**
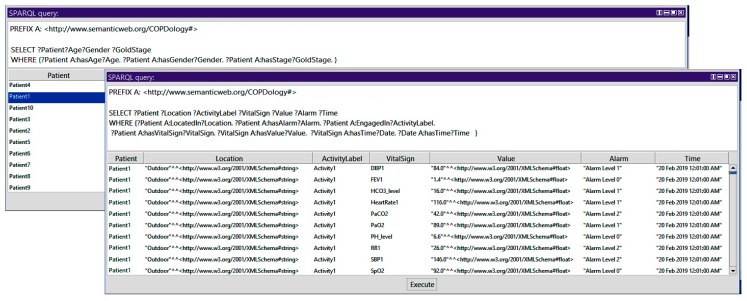
Query 1 and result.

In the query presented in the Figure 17, we display the identifier of all patients and their corresponding information. We use the triple pattern of Patient hasAge Age, Patient hasGender Gender, and Patient hasStage GoldStage. We use the SELECT query to get the information of the patient identifier, age, gender, and stage. In a similar way, we used the triple pattern of Patient LocatedIn Location, Patient hasAlarm Alarm, Patient EngagedIn ActivityLabel, Patient hasVitalSign VitalSign, HasValue Value, VitalSign hasTime Date, and Date hasTime Time. We use the SELECT query to get the information of location, activity, vital, signs, value, alarm, and time.

*Query 2: Show the Suggestions of All Calculated Levels of IAQI*

**Figure 18:**
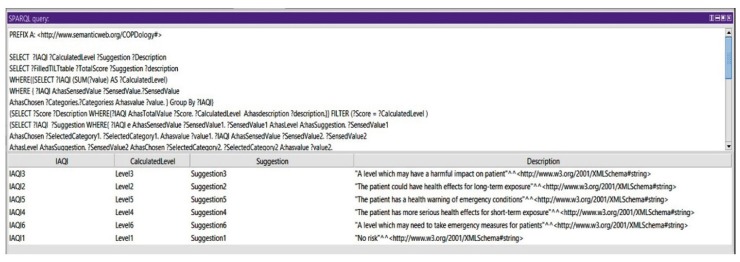
Query 2 and result.

Based on the indoor air quality index, we could query for the suggestions of all the IAQI levels. This query could show an aspect of the system in monitoring the environmental conditions. Figure 18 shows the calculated levels of IAQI and their corresponding suggestions.

*Query 3: Show Risk Scales*

**Figure 19:**
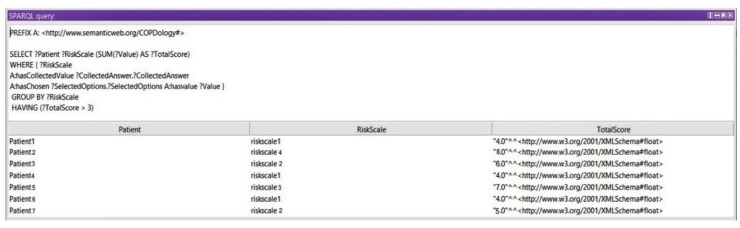
Query 3 and result.

In this query, we display the medical assessment of the patient at a certain moment based on the Ottawa COPD Risk Score. Any value between 3 and 9 could reflect a dangerous situation for a patient. In this query we get the risk of exacerbation after calculating the total scores of signs and symptoms. Based on the grouped levels and the total calculated score, we use a subquery to query the risk scale of the queried total scores (see Figure 19).

*Query 4: Show the Risk of Hypoxia at High Altitude Exposure*

**Figure 20:**
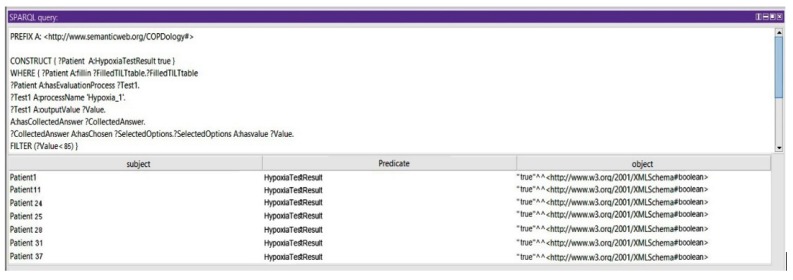
Query 4 and result.

In this query, we show that the patient could be prone to consequences of the initial exposure to high altitude: if the partial pressure and concentration of the oxygen are less than 50 mmHg and 85%, respectively, the patient cannot travel without supplemental oxygen (see Figure 20).

## 7. Results

The proposed ontology is suitable for two main scenarios. First, a typical home-based telemonitoring scenario that can provide self-management service by observing physiological data regarding the general health of individuals, as well as better information about the indoor air parameters such as temperature, humidity, pollutant gases, dust, and vapor, which are considered triggering factors for COPD. The second scenario is designed for tracking patients outside the home. In the outdoor monitoring area, the system gathers contextual information or environmental stimulus that could influence the biomedical parameters.

To illustrate the use of the model, we will perform some experiments. Let us consider the scenario of a COPD patient being remotely monitored after diagnosis. The patient’s vital signs, activity, and environmental parameters are continuously monitored. The patient’s profile is shown in Table 7.

The requirements for the supervision configured by the medical rules are depicted in each experiment. The parameters to be monitored correspond to specific limits. All these settings are described as parameters, constraints, and alarms. The defined alarms are associated with constraints configured for each profile. The activation of any of the alarms would alert both the patient and the physician; an earlier alert should be sent to the patient and pneumologist, with a visible notification and audible warning. These experiments allow for analyzing the fulfillment of the medical rules and services provided by varying both the vital signs and the activities of the patient. These experiments are classified as follows.

*Experiment 1—The Main Objective of This Experiment Is to Examine the Ability of the Ontology to Detect Abnormal Changes in Physiological Parameters.*

The alarms and constraints that control these changes are listed in the Table 8.

**Figure 21:**
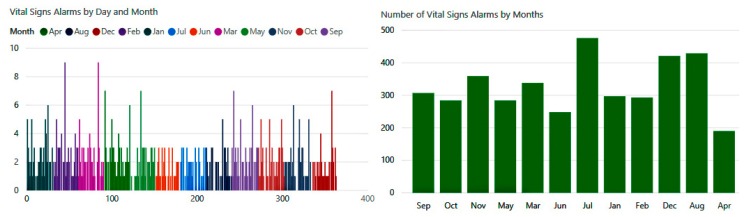
Number of alarms when the vital signs are abnormal.

The number of biometric alerts in Figure 21 is illustrated on a monthly basis. The experiment reveals that there were hundreds of changes in vital signs detected by our system during this year. We observe that the highest rates occurred during the months of July, August, and December, when the system detected about 1300 cases of abnormal conditions.

*Experiment 2—The Aim of This Experiment Is to Determine the Protective Capacity against Indoor and Outdoor Pollutants and Guarantee the Fulfillment of the Pollution Rules Related to the Patient Profile.*

As demonstrated in the Table 9 below.

**Figure 22:**
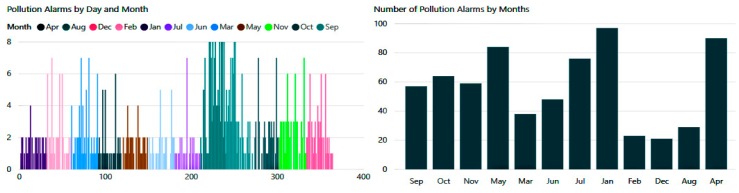
Number of alarms when the pollution index is dangerous.

As shown in Figure 22 below, pollution hazards in both internal and external spaces were also numerous. According to the conducted experimentation, the patient received between two and eight notification alerts per day, which would reduce the incidence of exacerbations significantly.

*Experiment 3—The Main Objective of This Experiment Is to Show the Importance of the Recommendations when Patients Are Not Protected by Weather Rules.*

Table 10 provides some of the alarms and constraints for climatic conditions that suit a specific profile of copd patients.

**Figure 23:**
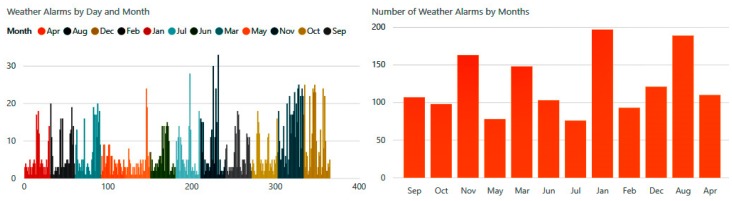
Number of alarms during extreme weather.

Daily weather alerts were distributed between alarms related to temperature, humidity, atmospheric pressure, wind, and precipitation. Figure 23 shows the number of climatic notifications received by the patient during the different months of the year.

*Experiment 4—The Purpose of This Experiment Is to Evaluate the Role of Activities Rules.*

Table 11 provides some of the alarms and constraints for everyday activities that suit a specific profile of copd patients.

**Figure 24:**
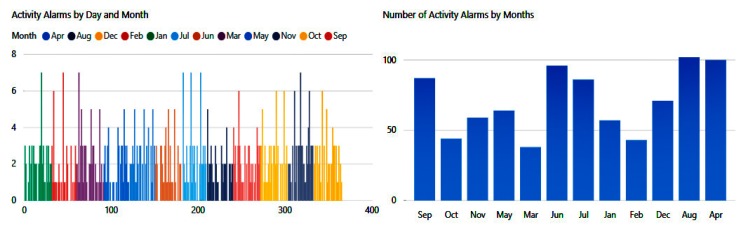
Number of alarms related to activities.

In Figure 24, we can see the number of risks that the patient may experience because of his involvement in some unsafe activities or nonadherence to proper nutrient intake.

The presented simulations have handled a great amount of data (525,600 records) of possible situations. The generated alarms have been classified into four main categories: vital signs, activity, pollution, and weather. The system applies continuous monitoring and detects a total of 3962 abnormal situations. Table 12 provides detailed information about the number of alarms during the different seasons.

## 8. Evaluation and Validation

The existing systems sought to promote their products without giving much attention to context design and performance; instead, their research focused on technology as a practice sustained by patients. It is important that the systems being used are supported by clinical practice guidelines and protocols to maintain consistency and minimize medical errors. Without a well-validated framework, the healthcare system will fail to provide any kind of protection or self-management services. Accuracy remains an important challenge for the alerts generated by the decision support systems. Practically, it is not known exactly how changes in physical parameters would affect the accuracy of the predefined alerts and their impact on patient outcomes. Improving the accuracy would enhance the overall performance of the telemonitoring systems and thus substantially reduce the disease burden.

As previously mentioned, the problem of telemonitoring systems was the impact of commercial and industrial aspects on research, which pushed hard towards blind adoption without a rigorous evaluation of the proposed designs. This lack of credible evaluation raises many questions about the feasibility and efficacy of this technology. This section will explore the performance of our ontology using a confusion matrix. Performance measurement quantifies accuracy, sensitivity, specificity, and the probability of predicting a dangerous change in physiological parameters in COPD patients. The purpose of this evaluation is to measure the accuracy of the alarms at the technical and clinical levels. A master file was created in MS Excel® that contains 1200 patient records, biometric readings for each patient (body temperature, diastolic blood pressure (DBP), systolic blood pressure (SBP), heart rate, partial pressure oxygen (PaO_2_), oxygen saturation (SpO_2_), partial pressure carbon dioxide (PaCO_2_), oxygen consumption (VO_2_), respiration rate, pH levels, concentration of hydrogen carbonate in the blood (HCO_3_), and FEV1 have been extracted from the results inferred from ontology in different scenarios. This information was presented to physicians in the following format (see Table 13):

The data are used to calculate the confusion matrices for the physician’s report outcomes. There are four possible outcomes: true positive (TP), true negative (TN), false positive (FP), and false negative (FN). The confusion matrix contains information about the predicted classifications identified by our ontology and the opinions of medical experts. The designation of the categories is as follows: (1) the alarm that is generated is based on a threshold set into the SWRL rules for each biometric parameter, (2) the values are interpreted within the ontology to identify when intervention is needed, (3) once a risk level is established the classifier is designated into one of four categories (TP, TN, FP, FN) based on physician’s report outcomes. Categories in this research are defined as follows:

1. TP is an alarm with a hospitalization.
2. FN has no alarm with a hospitalization.
3. TN has no alarm and no hospitalization.
4. FP has an alarm with no hospitalization.

The confusion matrices in Table 14 illustrated the results of the issued alarms based on the physician’s report outcomes. The next Table 15 summarizes the performance of our ontology include accuracy, sensitivity, specificity, the FPF, representing 1-specificity, FNF, PPV, and NPV.

The results indicate that our model reaches an accuracy of 88% in a set of 1200 clinical cases. Sensitivity and specificity have high values, denoting the ability of the ontology to detect warning signs. The positive predictive value (PPV) is defined as the probability of intervention for positive test results, while the negative predictive value (NPV) describes the probability of being healthy despite negative test results.

## 9. Conclusions and Limitations

The proposed approach will provide significant potential to address some of the current COPD challenges because it establishes new obligations that will limit many potential hazards at different levels, both physiological and environmental. The presented model provides automated alarm generation algorithms for the telemonitoring of COPD patients. The ontology can recognize any important changes in biometrics and environment based on a personalized threshold. The protection process aims to adjust the thresholds around the normal state to avoid exacerbation triggers. Our findings proved that dynamic thresholds can enhance existing telemonitoring systems and make a valuable contribution to identifying the health status of COPD patients. Three main conclusions can be drawn from this work. Firstly, an ontology-based system can provide a more efficient way to deal with medical data. Secondly, adding an SWRL layer of experts’ rules on top of OWL can handle various types of context and suggest reliable recommendations. Thirdly, the results support the importance of context where it demonstrates that context variables have a strong influence on the accuracy of decisions. This work has some limitations at the decision-making and implementation levels. The central reasoning engine is based on rules defined by experts, thus in a case where pneumologists fail to mention some risk scenarios, the system is unable to detect abnormal conditions directly. Moreover, the proposed system is considered relatively more complex than the traditional system because it must deal with large ontologies and relational databases simultaneously, which may reduce the computing performance in terms of response time. Further research is needed before the practical application of this approach. This study will have to be evaluated through real implementation and a crossover trial to assess its success and effectiveness.

## Figures and Tables

**Table 1:** Heart rate variation.

Male Patients, *n* = 1370	Stage	Age 40‒50 *n* = 223	Age 50‒60 *n* = 295	Age 60‒70 *n* = 307	Age 70‒80 *n* = 280	Age 80‒90 *n* = 265	*p*-Value
Principal Classification	Stage I	72.6 ± 12	69.2 ± 11	70.1 ± 8	68.70 ± 7	67.20 ± 9	<0.005
	Stage II	74.6 ± 13	72.3 ± 12	71.3 ± 11	70.3 ± 10	69.3 ± 12	<0.005
	Stage III	77.5 ± 13	75.2 ± 12	74.2 ± 11	73.6 ± 11	72.6 ± 10	<0.005
	Stage IV	84.9 ± 14	82.2 ± 13	81.2 ± 12	80.2 ± 10	79.2 ± 11	<0.005
Changes in principal classification, Standard Variation, Odds Ratio, Confidence Interval and *p*-Value							
**Influencing factors**	**Subfactors**	**Mean**	**Change (SD)**		**OR**	**CI 95%**	***p*-Value**
Smoker		80.1	6.6 ± 10		0.573	0.43‒0.76	<0.001
BMI	Underweight	-	-		-	-	NS
	Normal	-	-		-	-	NS
	Overweight	-	-		-	-	NS
	Obese	82.6	9 ± 4		0.673	0.51‒0.88	0.004
Inhaler Medication		80.7	7 ± 3		1.512	1.15‒1.98	0.003
Comorbidities	CHF	83.8	9.6 ± 10		0.43	0.28‒0.65	<0.001
	HBP	-	-		-	-	NS
	Anemia	79.5	7 ± 10		0.954	0.89‒0.97	<0.001
	IHD	-	-		-	-	NS
	pH	84.3	10 ± 13		0.776	0.58‒0.98	0.0037
	GERD	-	-		-	-	NS
	Asthma	78.8	8 ± 10		0.46	0.30‒0.71	<0.005
Exercise	Sedentary	76.3	3 ± 10		1.22	0.75‒1.9	NS
	Light	85.5	9 ± 15		0.931	0.87‒0.99	0.0035
	Moderate	125.6	29 ± 18		1.007	0.99‒1.006	<0.001
	Vigorous	145.3	43 ± 25		0.895	0.87‒0.91	<0.001

**Table 2:** Dataset: examples of activities.

Date	Start Time	End Time Activity	Activity
Day 1	12:00	12:14	Sitting
Day 1	12:15	12:30	Walking
Day 1	12:31	12:34	Standing
Day 1	12:35	13:15	Driving
Day 1	13:16	13:40	Sitting
……	…….	……	…….
Day 2	08:00	08:20	Running
Day 2	08:21	08:30	Walking

**Table 3:** Examples from the dataset that describes the outdoor environment.

Date	Time	Humidity	Temperature	WD_V	WD_UV	WSP_VE	WSPD_SC	Precipitation	Pressure
Day 1	12:00	83.5	−14.56	134.2	113.2	0.153	0.381	0.3	102.069
Day 1	12:01	83.9	−14.56	214.5	211.2	0.467	0.791	0.3	102.041
Day 1	12:02	82.8	−14.56	6.775	8.09	0.442	0.587	0.3	101.928
Day 1	12:03	80.1	−14.56	149.1	162.5	0.363	0.536	0.3	101.812
Day 1	12:04	80.6	−14.56	158	142.8	0.633	0.91	0.3	101.808
Day 1	12:05	83.8	−14.56	119.7	115.5	0.653	0.762	0.3	101.842
Day 1	12:06	88.1	−14.56	106.9	105	0.991	1.037	1.2	101.84
Day 1	12:07	89.7	−14.56	127.5	127.5	0.903	0.959	1.2	101.759
Day 1	12:08	88.4	−14.56	120.7	114.2	0.768	0.865	1.2	101.833
….	….	….	….	….	….	….	….	….	….
Day 2	1:01	73.9	−14.56	125.3	124.6	0.555	0.595	0	101.806
Day 2	1:02	78	−14.56	112.8	112	0.929	1.008	0	101.806

**Table 4:** Examples from the dataset that describes the outdoor environment.

Date	Time	AQHI	CO	H2S	NO	NO_2_	NOx	O_3_	PM10	PM2.5	SO_2_	TRS
Day 1	12:00	2.02	0.145315	0.24452	1.099305	2.848887	3.9425	32.393	23.61	0	0.2	2.1
Day 1	12:01	2.16	0.140258	0.47763	0.779582	1.397222	2.18083	34.953	17.4	0	0.1469	0.2
Day 1	12:02	2.24	0.140848	0.1424	0.403194	0.7675	1.170415	35.640	15.15	0	0.1283	0.2
Day 1	12:03	2.27	0.145591	0.10238	0.436111	1.249305	1.68387	35.233	19.2	0	0.1	0.2
Day 1	12:04	2.26	0.147459	0.10465	0.345694	1.589721	1.93777	34.836	30.5	3	0.1008	0.2
Day 1	12:05	2.27	0.153385	0.12705	0.575833	1.819721	2.39319	34.301	36.03	3	0.1057	0.2
Day 1	12:06	2.32	0.139437	0.19324	0.334583	1.494027	1.826805	34.689	26.33	5	0.1015	0.2
Day 1	12:07	2.45	0.137271	0.28494	0.207083	0.60736	0.821666	35.934	29.55	2	0.1	0.3
Day 1	12:08	2.59	0.122216	0.33706	0.25361	0.415555	0.6723	36.363	35.34	2	0.1	0.4
….	….	….	….	….	….	….	….	….	….	….	….	….
Day 2	1:01	2.78	0.119727	0.21297	0.143194	0.334443	0.4740	35.956	38.83	0	0.1	0.4
Day 2	1:02	2.92	0.116305	0.22772	0.276526	0.430415	0.711804	36.267	33.28	3	0.053	0.6

**Table 5:** Examples of data that describes the environmental conditions at home.

Date	Time	Indoor Temperature (°C)	Indoor Humidity (%)	CO_2_	VOC	PM2.5	PM10
Day 1	12:00	20.83	72.09	708	0.062	9	10.2
Day 1	12:01	21.01	70.95	694	0.062	10.1	10.9
Day 1	12:02	21.20	69.12	693	0.062	9.9	10.2
Day 1	12:03	21.37	68.83	692	0.062	9.6	9.6
Day 1	12:04	21.49	68.6	690	0.062	8.4	9.4
Day 1	12:05	21.66	68.31	690	0.062	6.8	6.8
Day 1	12:06	21.79	68.11	690	0.062	6.9	6.9
Day 1	12:08	22.90	67.79	691	0.062	7.3	8.1
….	….	….	….	….	….	….	….
….	….	….	….	….	….	….	….
Day 2	15:01	23.24	66.98	695	0.062	6.5	7.2
Day 2	15:02	23.36	66.63	695	0.062	7.2	7.6

**Table 6:** An example of a medical record.

**ID**	**Gender**			**Age**						**Height**			**Weight**					**BMI**													**Smoker**		**Comorbidities**											**Medication**	
##	Male			77						184			99					29.33													Yes		Anemia											Short-acting	
**Dyspnea (mMRC)**				**Gold stage**						**FVC (L)**			**FEV1**					**FEV1/FVC**							**FEV1/SVC**						**FEF 25‒75%**		**FEF50%**											**PEF**	**FET100%**
Mmrc2				2						2.33			1.01					43							40						0.40		0.46											3.86	9.61
**FET100%**		**FIF50%**								**FEF/FIF50**						**TLC**			**VC**								**IC**		**FRC PL**			**ERV**								**RV**			**RV/TLC**		**DLCO**
9.61		2.14								0.22						5.99			2.55								1.87		4.12			0.62								3.43			57		16.4
**Baseline Heart rate**									**H Reserve**						**Heart rate Max**									**HR (light exercise)**							**HR (moderate exercise)**											**HR (vigorous exercise)**			
60 beats/min									93 beats/min						153 beats/min									60‒97 beats/min							98‒120 beats/min											121‒130 beats/min			
**Baseline temperature (T)**											**T (light exercise)**																	**T (moderate exercise)**													**T (vigorous exercise)**				
36.39 °C											36.95 °C																	37.26 °C													37.79 °C				
**Baseline SpO_2_**					**SpO_2_ (light exercise)**																		**SpO_2_ (moderate exercise)**											**SpO_2_ (vigorous exercise)**											
96.01%					95.21‒96.01%																		93.10‒95%											91.30‒92.6%											
**Baseline PaO_2_**								**PaO_2_ (light exercise)**													**PaO_2_ (moderate exercise)**																		**PaO_2_ (vigorous exercise)**						
78 mmHg								75‒82 mmHg													83‒88 mmHg																		89‒95 mmHg						
**Baseline PaCO_2_**								**PaCO_2_ (light exercise)**														**PaCO_2_ (moderate exercise)**															**PaCO_2_ (vigorous exercise)**								
39 mmHg								38‒41 mmHg														34‒39 mmHg															32‒39 mmHg								
**Baseline DBP**							**DBP (light exercise)**														**DBP (moderate exercise)**															**DBP (vigorous exercise)**									
75.9 mmHg							75‒78 mmHg														79‒82 mmHg															83‒90 mmHg									
**Baseline SBP**						**SBP (light exercise)**														**SBP (moderate exercise)**													**SBP (vigorous exercise)**												
120 mmHg						121‒140 mmHg														141‒145 mmHg													146‒155 mmHg												
**Baseline Respiration rate (RR)**														**RR (light exercise)**												**RR (moderate exercise)**									**RR (vigorous exercise)**										
14 R/min														14‒19 R/min												20‒30 R/min									31‒50 R/min										
**Baseline VO_2_**			**VO_2_ Reserve**									**VO_2_ Max**					**HR (light exercise)**													**HR (moderate exercise)**								**HR (vigorous exercise)**							
2.53			14.129									16.59					2.5‒6.34 mL/kg/min													6.4‒9.36 mL/kg/min								9.5‒11.79 mL/kg/min							

**Table 7:** Profile of the patient.

Patient Profile		
**Age: 51**	Baseline PaO_2_: 78	Baseline DBP: 75
**Gender: Male**	Baseline PaCO_2_: 39	Baseline FEV1: 1.73 L
**BMI: 23**	Baseline SpO_2_: 96	Baseline VO_2_: 2.53
**GOLD Stage: I**	Baseline Heart rate	Baseline pH: 7.3
**Comorbidities: GERD**	Baseline Temperature: 36.95	Baseline HCO_3_: 25
**Baseline Dyspnea (MMRC): MMRC2**	Baseline Respiration rate: 15	
**Smoker: No**	Baseline SBP: 115	
**Medication: short-acting beta-agonist (SABA) and long-acting beta-agonist (LABA)**		

**Table 8:** Biomarker alarms and constraints for a specific profile.

Parameter	Constraint	Alarm
Blood Pressure: Diastolic Pressure (DBP) Systolic Pressure (SBP)	Light activity	110 < SBP > 130 AND 70 < DBP > 78 mmHg
	Moderate activity: 20 min	110 < SBP > 146 AND 70 < DBP > 78 mmHg
	Vigorous activity: 15 min	110 < SBP > 168 AND 70 < DBP > 78 mmHg
Heart Rate (HR)	Light activity	77 bpm < HR > 113 bpm
	Moderate activity: 1 h	80 bpm < HR > 144 bpm
	Vigorous activity: 15 min	85 bpm < HR > 160 bpm
SpO_2_	Light activity	95% > SpO_2_ > 97%
	Moderate activity: 30 min	94% > SpO_2_ > 96%
	Vigorous activity: 15 min	91% > SpO_2_ > 96%
Temperature	Light activity	37 °C > Temp > 38.1 °C
	Moderate activity	37.8 °C > Temp > 38.4 °C
	Vigorous activity: 20 min	38 °C >Temp > 38.8 °C
PaO_2_	Light activity	75 mmHg > PaO_2_ > 80 mmHg
	Moderate activity: 1 h	78 mmHg > PaO_2_ > 85 mmHg
	Vigorous activity: 15 min	80 mmHg > PaO_2_ > 98 mmHg
PaCO_2_	Light activity	36 mmHg > PaCO_2_ > 42 mmHg
	Moderate activity: 1 h	35 mmHg > PaCO_2_ > 40 mmHg
	Vigorous activity: 15 min	30 mmHg > PaCO_2_ > 36 mmHg
Respiration Rate (RR)	Light activity	14 br/min > RR > 20 br/min
	Moderate activity: 1 h	15 br/min > RR > 35 br/min
	Vigorous activity: 15 min	20 br/min > RR > 50 br/min
FEV1	Light activity	2.5 L > FEV1 > 2.7 L
	Moderate activity: 30 min	2.4 L > FEV1 > 2.7 L
	Vigorous activity: 10 min	2.2 L > FEV1 > 2.7 L
VO_2_	Light activity	2.5 mL/kg/min > VO_2_ > 6.5 mL/kg/min
	Moderate activity: 20 min	3.09 mL/kg/min > VO_2_ > 9.7 mL/kg/min
	Vigorous activity: 10 min	4.3 mL/kg/min > VO_2_ > 11.8 mL/kg/min
pH Level	Light activity	7.18 > pH > 7.34
	Moderate activity: 1 h	7.05 > pH > 7.24
	Vigorous activity: 1 h	6.93 > pH > 7.12
HCO_3_	Light activity	20 > HCO_3_ > 30
	Moderate activity: 1 h	16 > HCO_3_ > 26
	Vigorous activity: 1 h	15 > HCO_3_ > 24

**Table 9:** Pollutants alarms and constraints for a specific profile.

Parameter	Constraints	Alarm
Carbon monoxide (CO)	Exposure Limit: 1 h	CO > 2 ppm
Formaldehyde (HCHO)	Exposure Limit: 1 h	HCHO > 0.04 ppm
Volatile organic compounds (TVOC)	Exposure Limit: 1 h	TVOC > 0.9 ppm
Carbon dioxide (CO_2_)	Exposure Limit: 8 h	CO_2_ > 600 ppm
Particulate matter PM10	Exposure Limit: 24 h	PM10 > 60 µg/m^3^
Particulate matter PM2.5	Exposure Limit: 24 h	PM2.5 > 45 µg/m^3^
Ozone (O_3_)	Exposure Limit: 8 h	O_3_ > 0.03 ppm
Bacteria	No tolerance	Bacteria > 600 CFU/m^3^
Nitrogen dioxide (NO_2_)	Exposure Limit: 8 h	NO_2_ > 5 ppm
Sulfur dioxide (SO_2_)	Exposure Limit: 8 h	SO_2_ > 0.06 ppm
Hydrogen Sulfide (H2S)	Exposure Limit: 8 h	H2s > 1 ppm
Nitric oxide (NO)	Exposure Limit: 8 h	NO > 25 ppm
Nitrogen oxides (NOx)	Exposure Limit: 8 h	NOX > 10 ppm
Total reduced sulfur (TRS)	Exposure Limit: 4 h	TRS > 10 ppm

**Table 10:** Weather alarms and constraints for a specific profile.

Data	Constraint	Alarm
Indoor temperature	9 h in the bedroom	18 °C > Temperature > 18.5 °C
	Living room	Temperature > 21 °C
Indoor humidity	No additional condition	30% > Humidity > 50%
Indoor pressure	No additional condition	1013 kpa > Pressure > 1018 kpa
Outdoor humidity	Temperature < 30 °C	Humidity > 75%
Outdoor temperature	Hot weather: 30 min	Temperature > 27 °C
	Cold weather: 30 min	Temperature < 14 °C
	Very cold weather: 15 min	Temperature < 5 °C
Outdoor pressure	15 °C < Outdoor Temperature < 25 °C	Pressure < 89.325 kpa
Windspeed	Exposure limit: 15 min	WS > 12 mph
	Exposure limit: 1 h	WS > 7 mph
Precipitation	Rainfall	PRF > 0.2 cm
	Snowfall	PSF > 5 cm

**Table 11:** Activities alarms and constraints for a specific profile.

Data	Constraint	Alarm
**Aerobic Training (AT)**	Endurance time: 30 min	If AT > 30 min
**Travel by airplane**	Hypoxia altitude simulation test (HAST)	SpO_2_ < 92% SpO_2_ < 84% with 6MWT PaO_2_ < 50 mmHg
**Mountain trip**	-	Altitude > 1050 m
**Food**	Sodium: 24 h	Sodium > 180 mg
	Fructose: 24 h	Fructose > 25 g
	Glucose: 24 h	Glucose > 20 mg
	Calcium: 24 h	CA < 1200 mg
	Vitamin D: 24 h	800 IU > Vitamin D > 2000 IU
	Vitamin C: 24 h	Vitamin C > 1000 mg
	Vitamin A: 24 h	Vitamin A > 850 mcg
	Vitamin E: 24 h	Vitamin E > 900 IU
	Vitamin B12: 24 h	Vitamin B12 > 2.2 mcg
	Iron: 24 h	Iron > 35 mg
	Zinc: 24 h	Zinc > 40 mg
	Magnesium: 24 h	Magnesium > 435 mg
	Carbohydrate: 24 h	Carbohydrate > 380 g
	Protein: 24 h	Protein > 80 g
	Fat: 24 h	Fat > 75 g
	Fiber: 24 h	15 g > Fiber > 30 g

**Table 12:** Total number of alarms.

Factor	Winter Exceeded Thresholds	Spring Exceeded Thresholds	SummerExceeded Thresholds	AutumnExceeded Thresholds
**Vital signs**	114	98	188	166
**Weather**	356	340	437	480
**Pollution**	298	200	391	377
**Activity**	160	84	196	41
**Total**	928	722	1212	1064

**Table 13:** Patient evaluation form.

**Profile**					Current Activity	**Vital signs**			
Age	Sex	BMI	Stage	Comorbidity		Temp	DBP	SBP	HR
									
**Vital signs**									
PaO_2_	PaCO_2_	SpO_2_	RR	PH	HCO_3_	FEV1	FVC	VO_2_	
								
**Physician’s Report**									


**Table 14:** The confusion matrix.

Ontology Recommendation	Physician’s Recommendation	TP	FP	TN	FN
**Hospitalization alarm**	Hospitalization	1			
**Hospitalization alarm**	No hospitalization		1		
**No hospitalization alarm**	No hospitalization			1	
**No hospitalization alarm**	Hospitalization				1

**Table 15:** Standard reference and results for the clinical decision support model.

Index	Intervention Present	Intervention Absent	Total
**Index test positive**	True positive (TP) = 512	False positive (FP) = 88	TP + FP = 600
**Index test negative**	False negative (FN) = 56	True negative (TN) = 544	TN + FN = 600
**Total**	TP + FN = 568	TN + FP = 632	
1. **Accuracy (AC) = (TP + TN)/(TN + FN + FP + TP) = 88%**			
2. **Sensitivity = (TP)/(TP + FN) = 91.14%**			
3. **False Negative Fraction (1-Sensitivity) = (FNF) = FN/(TP + FN) = 9.85%**			
4. **Specificity = (TN)/(TN + FP) = 86.07%**			
5. **False Positive Fraction (1-specificity) = (FPF) = FP/(TN + FP) = 13.92%**			
6. **Positive predictive value (PPV) = (TP)/(TP + FP) = 85.33%**			
7. **Negative predictive value (NPV) = (TN)/(FN + TN) = 90.66%**			
8. **F1 = ** ${\left(\frac{Recall^{-1}+\;Precision^{-1}}{2}\right)}^{-1}=$ **88.13%**			
